# Green Synthesis and Characterization of ZnO Nanoparticles Using *Pelargonium odoratissimum* (L.) Aqueous Leaf Extract and Their Antioxidant, Antibacterial and Anti-inflammatory Activities

**DOI:** 10.3390/antiox11081444

**Published:** 2022-07-26

**Authors:** Ahmed S. Abdelbaky, Taia A. Abd El-Mageed, Ahmad O. Babalghith, Samy Selim, Abir M. H. A. Mohamed

**Affiliations:** 1 Department of Biochemistry, Faculty of Agriculture, Fayoum University, Fayoum 63514, Egypt; 2 Department of Soil and Water, Faculty of Agriculture, Fayoum University, Fayoum 63514, Egypt; taa00@fayoum.edu.eg; 3 Department of Medical Genetics, College of Medicine, Umm Al-Qura University, P.O. Box 57543, Makkah 21955, Saudi Arabia; aobabalghith@uqu.edu.sa; 4 Department of Clinical Laboratory Sciences, College of Applied Medical Sciences, Jouf University, Sakaka 72388, Saudi Arabia; sabdulsalam@ju.edu.sa; 5 Department of Agricultural Microbiology, Faculty of Agriculture, Fayoum University, Fayoum 63514, Egypt; amh05@fayoum.edu.eg

**Keywords:** green synthesis, ZnO NPs, *characterization*, antioxidant, antibacterial, anti-inflammatory

## Abstract

Nanoparticles (NPs) exhibit distinct features compared to traditional physico-chemical synthesis and they have many applications in a wide range of fields of life sciences such as surface coating agents, catalysts, food packaging, corrosion protection, environmental remediation, electronics, biomedical and antimicrobial. Green-synthesized metal NPs, mainly from plant sources, have gained a lot of attention due to their intrinsic characteristics like eco-friendliness, rapidity and cost-effectiveness. In this study, zinc oxide (ZnO) NPs have been synthesized employing an aqueous leaf extract of *Pelargonium odoratissimum* (L.) as a reducing agent; subsequently, the biosynthesized ZnO NPs were characterized by ultraviolet-visible spectroscopy (UV-Vis), dynamic light scattering (DLS), Fourier transform infrared (FTIR) spectroscopy, X-ray diffraction (XRD), field emission scanning electron microscopy (FESEM) and energy-dispersive X-ray spectroscopy (EDX), high-resolution transmission electron microscopy (HRTEM) and selected area electron diffraction (SAED). Moreover, aqueous plant leaf extract was subjected to both qualitative and quantitative analysis. Antioxidant activity of ZnO NPs was assessed by DPPH assay, with varying concentrations of ZnO NPs, which revealed scavenging activity with IC_50_ = 28.11 μg mL^−1^. Furthermore, the anti-bacterial efficacy of the green synthesized ZnO NPs against four foodborne pathogenic bacterial strains was examined using the disk diffusion assay, and *Staphylococcus aureus* (ATCC 8095), *Pseudomonas aeruginosa* (ATCC10662) and *Escherichia coli* (ATCC 25922) were found to be the most sensitive against biosynthesized ZnO NPs, whereas the least sensitivity was shown by *Bacillus cereus* (ATCC 13753). The anti-inflammatory effect was also evaluated for both ZnO NPs and the aqueous leaf extract of *P. odoratissimum* through the human red blood cells (HRBC) membrane stabilization method (MSM) in vitro models which includes hypotonicity-induced hemolysis. A maximum membrane stabilization of ZnO NPs was found to be 95.6% at a dose of 1000 μg mL^−1^ compared with the standard indomethacin. The results demonstrated that leaf extract of *P. odoratissimum* is suitable for synthesizing ZnO NPs, with antioxidant, antibacterial as well as superior anti-inflammatory activity by improving the membrane stability of lysosome cells, which have physiological properties similar to erythrocyte membrane cells and have no hemolytic activity. Overall, this study provides biosynthesized ZnO NPs that can be used as a safe alternative to synthetic substances as well as a potential candidate for antioxidants, antibacterial and anti-inflammatory uses in the biomedical and pharmaceutical industries.

## 1. Introduction

Nanotechnology is one of the most quickly evolving fields, potentially forming and underpinning a wide range of technological and biotechnological advancements; as a result, it is seen as the century’s oncoming industrial revolution [1]. Nanotechnology has been used in different industrial and academic areas, including chemistry, agriculture, biology, medicine, electronics, information technology and physics [2,3,4]. Nanomaterials possess great potential in various fields of science due to their excellent physico-chemical and biological characteristics over bulk materials [5]. Nanoparticles (NPs) have the unique property of having a high surface-to-volume ratio [6], which means that they are more appropriate candidates for application-oriented performance (e.g., photocatalysis, cosmetics, gas sensing, energy reservoirs, electronics, packaging and environmental remediation) and encourages their incorporation into a wide range of commercial products, biotechnology and biomedical applications [7,8,9,10,11,12,13].

Among the large variety of NPs available, metal oxide (MO) NPs are thought to be the most promising because they have distinctive physical, chemical, and biological properties like solubility, chemical stability, and adhesiveness [8]. Additionally, the utilization of harmful compounds for reduction and as a capping agent in the nanoparticle synthesis process causes a variety of adverse effects on the flora life as well as the environment and the living system toxicity. As a result, plant extracts (PEs) are therefore a more promising tool for the easy synthesis of MO NPs through the green route, because this approach is eco-friendly, non-toxic, low cost, environmentally compatible and easy to apply. Additionally, the resultant particles are biocompatible and free of toxic stabilizers compared to classical chemicals. Basically, PEs contain a variety of active biomolecules that aid to reduce and stabilize NPs [6,12].

Zinc oxide (ZnO) is one of the very promising inorganic oxides that has recently attracted the attention of many scientists for the biosynthesis of NPs due to its unique properties and multiple applications such as drug delivery, solar cells, photocatalytic degradation and personal care products like sunscreens and cosmetics [14,15,16,17,18,19]. Based on earlier reports in the literature, ZnO NPs have been biosynthesized from several plant extracts such as *Cassia auriculata* [20], *Aloe vera* [13,21], *Duranta erecta* [22], *Cinnamomum verum* [23], *Bauhinia tomentosa* [24], *Vitex trifolia* [25], *Moringa oleifera* [26], *Azadirachta indica* [27,28], *Artocarpus gomezianus* [29] and *Olea europaea* [30]. In biological systems, the overproduction of highly reactive radical species (HRRS) causes oxidative stress, which has been observed in several diseases, i.e., cancer, diabetes, cardiovascular disease, and arthritis [31]. All biosystems depend heavily on antioxidants to function correctly. As a result, there is an urgent need to search for innovative and safe antioxidants produced from natural sources, which are more effective and less toxic. Additionally, the widespread use of antibacterial and anti-inflammatory drugs has caused resistance, the appearance of new pathogenic strains resistant to antibiotics [32] and chronic and acute toxicities in several human physiological systems, particularly the immune system. As a result, searching for new, effective antibacterial and anti-inflammatory drugs that can effectively combat drug-resistant bacteria is necessary and does not cause immunosuppression. Biosynthesized NPs have been proposed as an alternate potential approach to address these problems [33]. *Pelargonium odoratissimum* (L.) aqueous leaf extract (ALE) was utilized in the present study, for the biosynthesis of ZnO NPs as this is the first report on the use of this plant’s leaves for the green synthesis of NPs. *Pelargonium odoratissimum* (L.) known as “Apple Geranium” is a perennial and relatively flat-growing shrublet that belongs to the family Geraniaceae, very commonly grown locally in Egypt and is widely utilized for its health benefits [34]. Essential oils of *Pelargonium* spp. are in considerable demand in the pharmaceutical, perfumery, and cosmetic industries. Additionally, some reports revealed that essential oils obtained from a variety of *Pelargonium* spp. possess excellent antioxidant, antibacterial and antifungal properties [35,36,37,38]

The aerial parts of this *Pelargonium* spp. are used in traditional medicine for the treatment of wound healing, debility, gastrointestinal disorders (i.e., diarrhea and dysentery), hemorrhage, skin complaints, neuralgia and throat infections due to their various phytochemical constituents such as phenolics, flavonoids, terpenes, saponins and essential oils [39], which can contribute to their biological activities and facilitate the biosynthesis of NPs by employing them as reducing, capping and stabilizing agents.

Despite the widespread use of *Pelargonium* species as therapeutic agents, to date, there have been no data on their use for green synthesis of NPs, antioxidant, antibacterial and anti-inflammatory effects from *Pelargonium odoratissimum* leaf extract.

The aim of this study was to explore the application of *P. odoratissimum* ALE as a capping and reducing agent for the biosynthesis of ZnO NPs. The biosynthesized ZnO NPs were characterized and confirmed by various spectroscopic and microscopic techniques, i.e., UV-Vis spectroscopy, FTIR, XRD, DLS, HR-TEM, FE-SEM and EDX, in addition, to evaluate the antioxidant effects, as well as the antibacterial activities against some food-borne pathogens strains beside evaluating the anti-inflammatory activities of both ZnO NPs and the ALE of *P. odoratissimum*.

## 2. Materials and Methods

### 2.1. Chemicals

Gallic acid, rutin, 1,1-diphenyl-2-picrylhydrazyl (DPPH, ≥99%), Folin–Ciocalteu’s reagent, L-ascorbic acid (Sigma-Aldrich, St. Louis, MO 63103, USA), aluminum chloride anhydrous (Fluka, Buchs, Switzerland), sodium carbonate (>99%), zinc acetate dihydrate (Advent Chembio PVT. LTD, Mumbai, India), Luria-Bertani (LB) broth medium (Himedia, Mumbai, India) gentamycin (Tody Laboratories Int., 22nd Vadul Moldovei Street, Bucharest, Romania). All chemicals used in this study were of analytical grade.

### 2.2. Plant Collection and Processing

Fresh leaves of *P. odoratissimum* (L.) were collected from the Botanical Garden of Fayoum University, Fayoum, Egypt, in March 2021. The taxonomic identification of the plant was identified by Mrs. Therese Labib, Head of the Taxonomy specialists at El-Orman Botanical Garden, Cairo, Egypt. A voucher specimen with number 126 was deposited in the herbarium of the Biochem. Dept. Fac. Agric., Fym. Univ., Fym., Egypt. The leaves were completely air dried in the shade before being ground into a fine powder in a lab mill and sieved using a 24 mesh sieve. The powdered leaves were maintained in an air-tight container at room temperature (28 ± 2 °C) and kept away from light until use. 

### 2.3. Preparation of P. odoratissimum Leaf Extract

The air-dried powder (20 g) of *P. odoratissimum* leaves was taken and immersed in 400 mL of deionized water (dH_2_O). The extraction process was performed via the ultrasonic-assisted solvent extraction (UASE) method [40] by placing the conical flask in a Probe Sonicator homogenizer (Benchmark Scientific, USA, 150 W, 25 kHz) at room temperature (35 ± 2 °C) for 30 min. The solvent (d.H_2_O) and powder layer were filtered using muslin cloth first and then Whatman filter paper No.1. The filtrate solution of *P. odoratissimum* leaf extract was kept in a refrigerator to be utilized for further use.

### 2.4. Qualitative Phytochemical Screening

The detection of various phytoconstituents present in the ALE of *P. odoratissimum* was carried out using the standard phytochemical methods [41,42,43].

### 2.5. HPLC-Analysis

The HPLC analysis was carried out using an Agilent 1260 series. The separation was performed using Eclipse C18 column (4.6 mm × 250 mm i.d., 5 μm). The mobile phase consisted of water (A) and 0.05% trifluoroacetic acid (TFA) in acetonitrile (B) at a flow rate of 0.9 mL/min. The mobile phase was programmed consecutively in a linear gradient as follows: 0 min (82% A); 0–5 min (80% A); 5–8 min (60% A); 8–12 min (60% A); 12–15 min (82% A); 15–16 min (82% A) and 16–20 (82%A). The multi-wavelength detector was monitored at 280 nm. The injection volume was 5 μL for each of the sample solutions. The column temperature was maintained at 40 °C.

### 2.6. Estimation of Total Phenolic and Flavonoid Contents (TPC and TFC)

The determination of both TPC as mg gallic acid equivalents (GAE) mg GAE/g plant extract) and TFC as mg rutin equivalents (RE)/g plant extract were performed spectrophotometrically by the Folin-Ciocalteu reagent [44] and aluminum chloride methods [45] respectively. 

### 2.7. Green Synthesis of ZnO Nanoparticles

**Figure 1:**
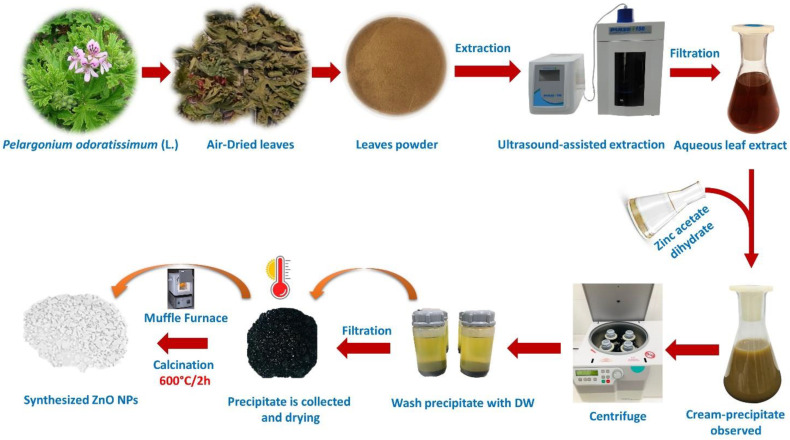
Represent (pictorial) the synthesis of ZnO NPs via *P. odoratissimum* ALE.

After heating twenty milliliters of *P. odoratissimum* leaf extract at 50 °C for 10 min, fifty milliliters of 0.1 M zinc acetate dihydrate (Zn(CH_3_COO)_2_·2H_2_O) (1.095 g of zinc acetate dihydrate was dissolved in 50 mL of d.H_2_O) was added drop-by-drop to it under stirring at 800 rpm that resulted in cream-colored zinc hydroxide precipitate formation. For the complete reduction in zinc hydroxide, the reaction mixture was left for 30 min. Then the precipitate was centrifuged (Sigma Laborzentrifugen 2k15, Osterode, Germany) at 16,000 rpm for 10 min at 4 °C by dH_2_O followed by ethanol repeatedly in order to remove the impurities. The precipitate was dried overnight in an oven at 100 °C. The obtained dried powder was calcined in a muffle furnace at 600 °C for 2 h and the white powder of ZnO NPs was obtained after calcination as shown in Figure 1. The resulted powder was used for characterization.

### 2.8. Characterization Methods of ZnO NPs

#### 2.8.1. UV-Vis Spectroscopy

In order to study the optical characteristics of green synthesized ZnO NPs, a known amount of ZnO NPs (0.05 g) was dispersed in 5 mL of ethanol (96%). The absorption spectrum was recorded by using a UV-Vis (U-2900) double beam spectrophotometer (Hitachi, Tokyo, Japan) in between a wavelength scan of 200–800 nm.

#### 2.8.2. Dynamic Light Scattering (DLS)

A particle size analyzer (Zetasizer V 2.2, Worcestershire, Malvern, UK) was utilized to determine the particle size distribution (PSD) of ZnO NPs obtained using ALE. The zeta potential of ZnO NPs was carried out in the water as a dispersant through a Zeta sizer (V 2.3, Worcestershire, Malvern, UK) to identify the stability of the synthesized NPs.

#### 2.8.3. Fourier Transform Infra-Red Spectroscopy (FTIR)

FTIR analysis (Bruker, Berlin, Germany) was employed to identify the functional groups (FGs) involved in biosynthesized ZnO NPs. At a wavelength of 4000–400 cm^−1^, the FTIR spectra were scanned with a resolution of 4.0 cm^−1^.

#### 2.8.4. X-ray Diffraction (XRD)

The crystalline structure of ZnO NPs was analyzed by an X-ray diffractometer (Bruker D8 DISCOVER, Bruker, Germany) with Cu-Kα radiation (λ = 1.54060 Angstrom). The relative intensity data were collected over a 2θ range of 5°–80°, 2θ values and relative intensities (I/Io) were determined from the chart, and the minerals of core materials were identified with JCPDS carts.

#### 2.8.5. Field Emission-Scanning Electron Microscopy (FE-SEM)

The topography and surface morphology of the biosynthesized ZnO NPs were examined using FE-SEM (Carl- ZEISS Sigma 500 VP, Sigma, Osterode, Germany) equipped with an energy dispersive X-ray spectrometer (EDX, Bruker, Germany) for the element composition present in the powder of ZnO NPs. A portion of the sample was set on a carbon-coated copper (CCC) grid, and the film on the FE-SEM grid was then dried by fixing it under gold for 5 min. 

#### 2.8.6. High-Resolution Transmission Electron Microscopy (HRTEM)

The shape and size distribution of powdered ZnO NPs were studied by using HRTEM (JEM-2100, JEOL, Tokyo, Japan) at an accelerated voltage of 200 kV.

### 2.9. Estimation of Antioxidant Activity—DPPH Radical Scavenging Activity

The ability to scavenge the free radical DPPH of the ALE of *P. odoratissimum*, biosynthesized ZnO NPs and standard L-ascorbic acid at different concentrations ranging from 3125–100 μg mL^−1^ were performed using the Brand-Williams et al. method [46]. Briefly, 2 mL of the DPPH solution (Sigma-Aldrich, 3050 Spruce Street, St. Louis, MO 63103, USA) (25 mg L^−1^ in methanol) was added to 0.1 mL of different concentrations of each sample and standard L-ascorbic acid (3125–100 μg mL^−1^). After shaking vigorously for 1 min, the reaction mixture was maintained in the dark for 30 min at room temperature (35 ± 2 °C) and the absorbance was recorded at 517 nm using the U-2900 UV-Vis double beam spectrophotometer (Hitachi, Tokyo, Japan). Each measurement was taken in three replications. The free radical scavenging activity (FRSA) of each sample was expressed as percent inhibition of DPPH free radical and was calculated as:

% inhibition (% Anti-radical activity) = [(A _control_ − A _sample_)/A _control_] × 100, where A is the absorbance. The IC_50_ values were measured from the relationship curve of FRSA versus concentrations of the respective sample curve. 

### 2.10. Estimation of Antibacterial Activity

#### 2.10.1. Bacteria Strains

The antibacterial effect of the biosynthesized ZnO NPs with *P. odoratissimum* ALE was established against two Gram-positive bacteria (GPB), *Bacillus cereus* (ATCC13753) and *Staphylococcus aureus* (ATCC8095), and two Gram-negative bacteria (GNB), *Escherichia coli* (ATCC25922) and *Pseudomonas aeruginosa* (ATCC10662). These four strains were acquired from the Microbiol. Dept., Fac. Agric., Fym. Univ., Egypt. The bacterial strains used were maintained in the Luria–Bertani (LB) agar at 30 °C for 24 h and then kept at 4 °C in a refrigerator. During this study, LB media was used for all bacterial cultures. 

#### 2.10.2. Antibacterial Assay

The antibacterial effect against the examined bacterial strains was determined using the agar disc diffusion method (ADDM) described by Bauer et al. [47]. In this method, three different ZnO NPs concentrations (10, 20 and 30 μg mL^−1^) and ALE (20 μg mL^−1^) were dissolved in ethanol and then used to fill sterilized Whatman filter paper discs of approximately 40 μL with the proper volume containing the tested ZnO NPs concentrations and ALE and left to totally dry. A disc containing only solvent was used as a negative control and a disc containing zinc acetate dihydrate was employed. A positive control gentamicin (10 μg mL^−1^) was used. Overnight bacterial cultures were prepared in LB broth for obtaining tested bacterial suspensions for the assay. The discs were then placed on the plates having the tested bacterial cultures and diluted to obtain about 1 × 10^−7^ colony-forming unit (CFU). The inoculated plates were incubated at 37 °C for 24 h and then the activity was assayed by measuring the inhibition diameter in millimeters (mm). All tests were performed in triplicate.

### 2.11. Estimation of Anti-inflammatory Activity

The human red blood cells (HRBCs)-membrane stabilization method (HRBCs-MSM) has been performed for the evaluation of in vitro anti-inflammatory activity according to the procedure outlined by Anosike et al. [48].

### 2.12. Statistical Analysis

All of the tests (antioxidant, antibacterial, and anti-inflammatory activity) were performed in triplicates, with the results provided as mean ± SD. Using the statistical software SPSS (SPSS version 21, IBM Corporation, Armonk, NY, USA), the statistical data were examined using the two-way ANOVA technique. The difference in significance was calculated at *p* < 0.05.

## 3. Results and Discussion

### 3.1. Qualitative Phytochemical Screening (QPS)

The results of the QPS of *P. odoratissimum* ALE are summarized in (Table 1), which displays the existence of saponins, phenolics and tannins, flavonoids, carbohydrates and/or glycosides and the absence of steroids, triterpenoids and alkaloids. These present compounds could be responsible for the bio-reduction of the metal salts into nanosize particles [49].

### 3.2. HPLC-Analysis

**Figure 2:**
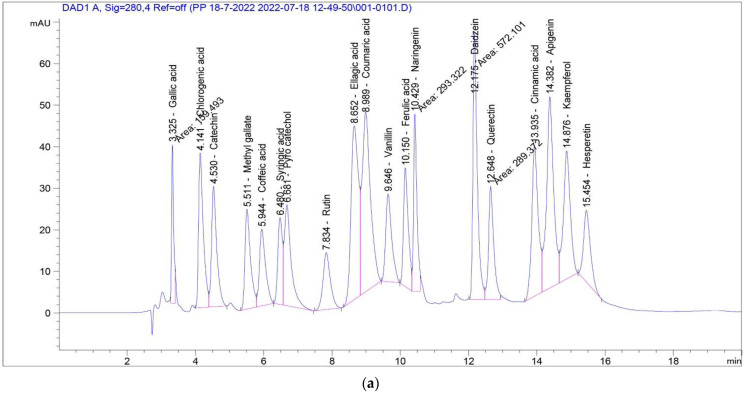

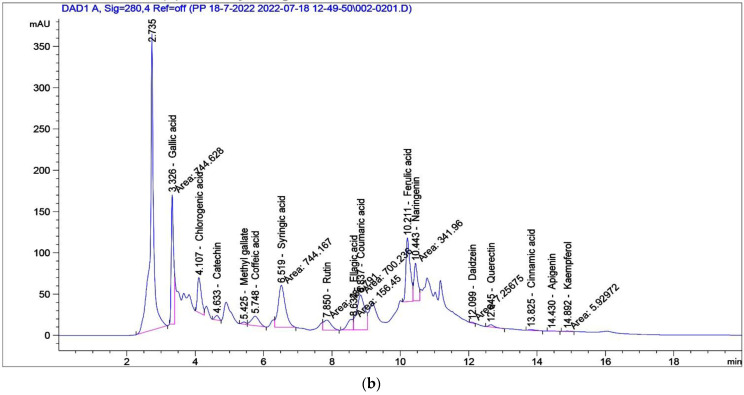
HPLC chromatogram: (**a**) standard polyphenolic compounds; (**b**) ALE of *P. odoratissimum*.

**Figure 3:**
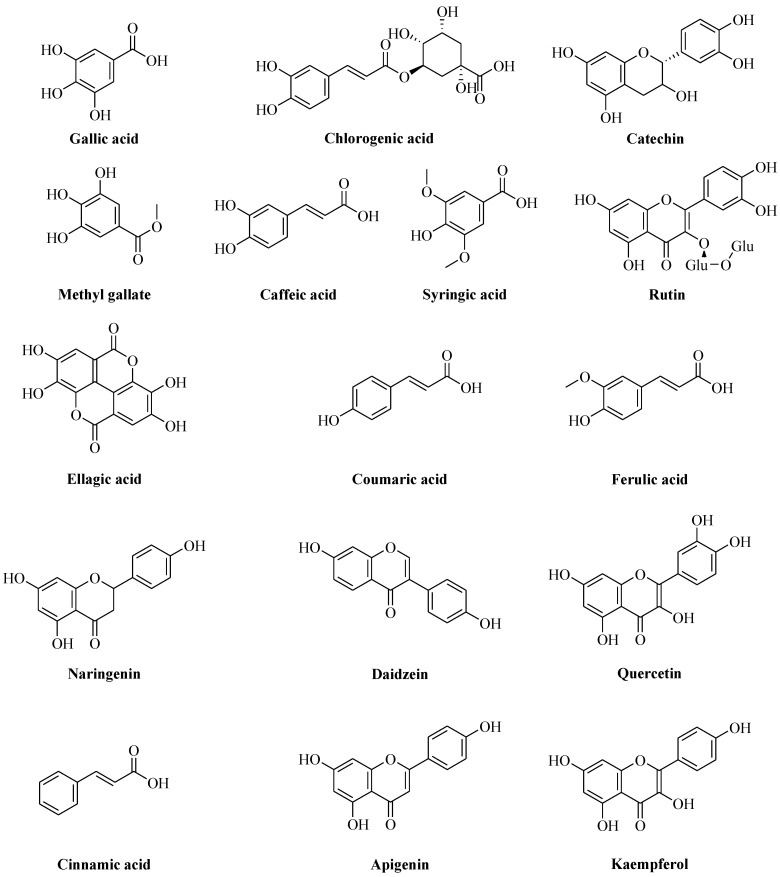
Chemical structures of polyphenolic compounds present in *P. odoratissimum* ALE.

HPLC analysis of ALE indicates the presence of sixteen phenolic compounds in appropriate amounts: Gallic acid, Chlorogenic acid, Catechin, Methyl gallate, Caffeic acid, Syringic acid, Rutin, Ellagic acid, Coumaric acid, Ferulic acid, Naringenin, Daidzein, Quercetin, Cinnamic acid, Apigenin and Kaempferol (Table 2 and Figure 2, respectively), that may be responsible for the bio-reduction of the metal salts into ZnO-nanosize particles. Additionally, Gallic acid, Syringic acid, Chlorogenic acid, Ferulic acid, Naringenin, Ellagic acid, Rutin and Coumaric acid were found to be highly prevalent among several significant phenolic components identified. Both phenolic acids and flavonoids are known to be potent hydrogen donors [50], which are responsible for a variety of biological activities because of their functional (carboxyl and hydroxyl) groups. The amounts (µg/g) and structures of polyphenols are illustrated in Table 2 and Figure 3, respectively.

### 3.3. Characterization of ZnO NPs

#### 3.3.1. Visual Observation

**Figure 4:**
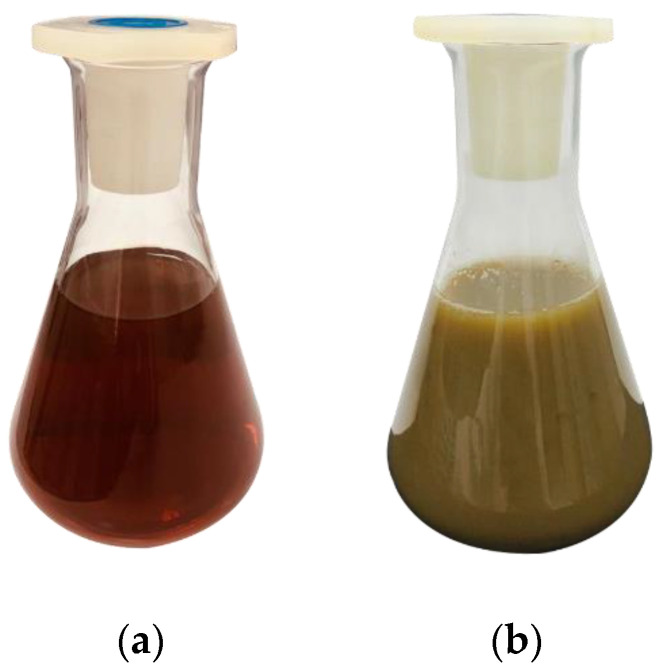
The visual observation of colour changes at 0 time (**a**) (*P. odoratissimum* ALE) and after 30 min (**b**) (*P. odoratissimum* ALE and (Zn(CH_3_COO)_2_·2H_2_O)).

The first essential indicator that confirms the biosynthesis of ZnO NPs is visual observation. When the Zn(CH_3_COO)_2_·2H_2_O, as a precursor for ZnO NPs, was added to the *P. odoratissimum* leaf extract, the color of the *P. odoratissimum* leaf extract was changed from light red to cream-colored precipitate (Figure 4). Similar color changes of synthesized ZnO NPs employing *Hibiscus subdariffa* leaf extract, from light red to cream-colored precipitate, were displayed by Bala et al. [16], confirming the biosynthesis of ZnO NPs.

#### 3.3.2. UV-Vis Spectroscopy

**Figure 5:**
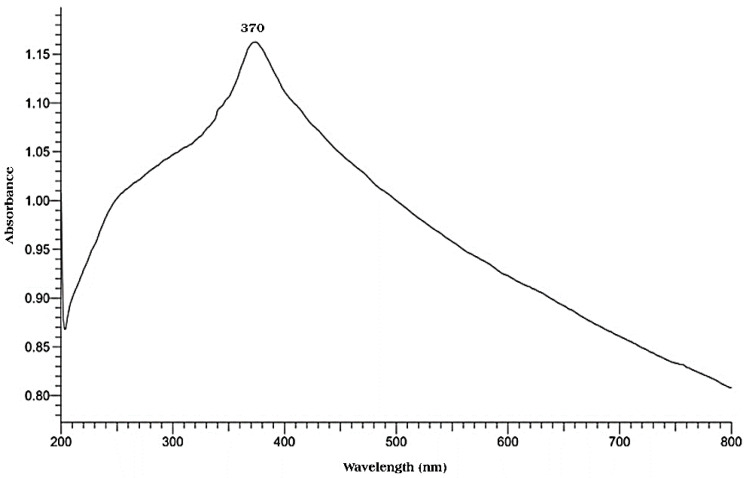
UV/Vis spectrum of ZnO NPs biosynthesized using *P. odoratissimum* ALE.

To confirm the synthesis of ZnO NPs, UV/Vis spectrophotometry was performed in order to examine the optical characteristics of green synthesized ZnO NPs using *P. odoratissimum* ALE. The UV-Vis spectrum recorded the maximum absorbance peak at 370 nm as shown in Figure 5, which verified the synthesis of ZnO NPs via *P. odoratissimum* ALE, which is consistent with earlier studies by Senthilkumar et al. [51], who examined the ability of *Tecona grandis* (L.) ALE to synthesize ZnO NPs with surface plasmon resonance (SPR) at 370 nm. Additionally, there are no other peaks recorded in the spectrum which means that the biosynthesized ZnO NPs are a pure product. Furthermore, the high absorption band seen at 378 nm might be attributed to ZnO’s inherent band-gap absorption caused by electron transitions from the valence band (E_V_) to the conduction band (E_C_) (O_2p_–Zn_3d_) [52,53]. The formula for calculating the energy bandgap (E_G_) of ZnO NPs was used as follows:

E_G_ = hc/λ (1)

Where h is Planck’s constant (6.626 × 10^−^^34^ Js), c is the velocity of light (3 × 10^8^ m/s) and λ is the wavelength (378 nm). In total, 3.28 eV was found to be the bandgap energy of ZnO. The significant UV absorption of ZnO NPs demonstrates the product’s suitability for a variety of medicinal applications, including sun-screen protectors and antibacterial ointments [54].

#### 3.3.3. Dynamic Light Scattering (DLS) 

**Figure 6:**
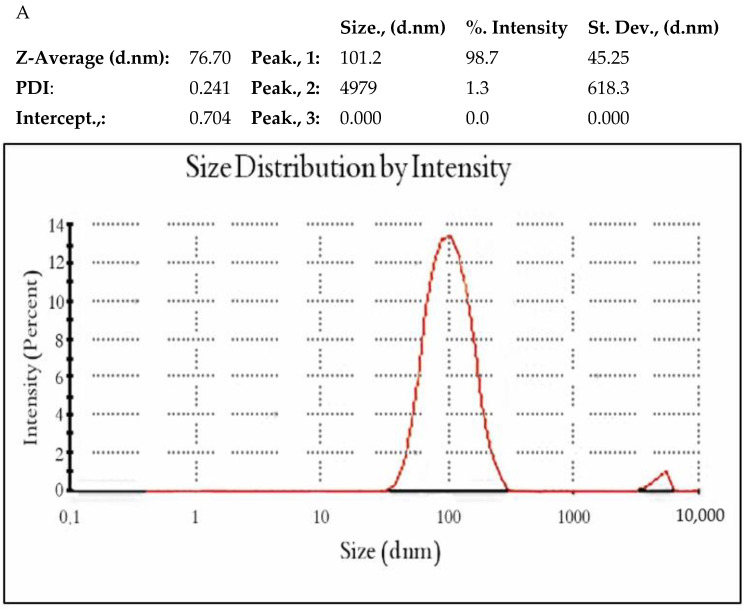

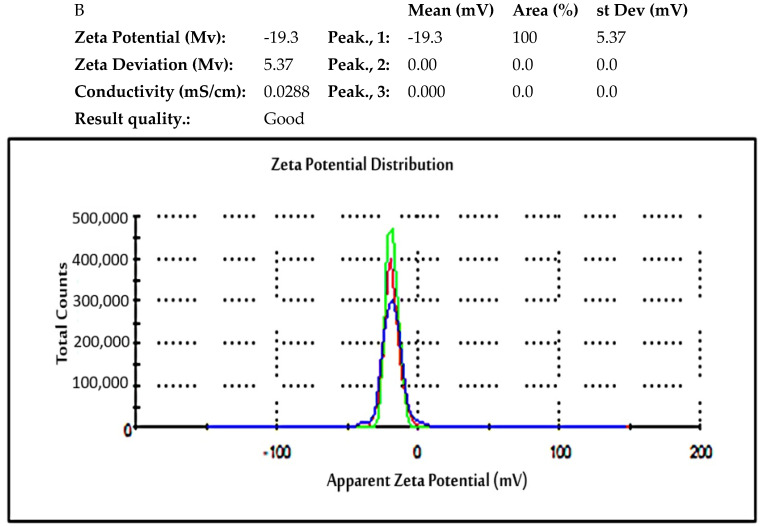
PSD (**A**) and ZP (**B**) of green synthesized *P. odoratissimum*-ZnO nanoparticles.

The Z-average diameter (nm) and PSD of the biosynthesized ZnO NPs were measured using the DLS technique. As shown in Figure 6A, the measurements demonstrated that the average size (nm) of the ZnO NPs with *P. odoratissimum* ALE was about 76 nm. The result obtained from the PSD profile of the ZnO nanoparticles revealed two notable peaks with intensities of 98.7% and 1.3%. Additionally, the ZnO NPs have a polydispersity index (PDI) of 0.241. This indicated that ZnO nanoparticles are very homogeneous and have a uniform size range [55]. This finding is completely compatible with Badran, Chen et al. and Putri et al. [56,57,58] who reported that PDI values of 0.3 and below are considered to be monodisperse. Because of the hydrodynamical shell, the DLS technique is known to produce significantly higher values than HRTEM size analyses. Additionally, the size of the hydrodynamical shell is influenced by particle structure, particle shape, and roughness [59].

The surface charges and stability of biosynthesized ZnO NPs have been assessed through zeta potential (ZP) analysis. The ZP graph of ZnO nanoparticles is presented in (Figure 6B). As shown in Figure 6B, the ZP was found to be −19.3 mV which indicates the potential stability of the examined NPs [51]. As a result, the reducing agents (i.e., phenolic and flavonoid components) found in the leaf extract (LE) are probably responsible for the negative charge potential of the produced ZnO NPs. It also confirms that the produced substance contains substantial electrostatic forces [60].

#### 3.3.4. FTIR Analysis of Biosynthesized ZnO NPs and *P. odoratissimum* ALE

**Figure 7:**
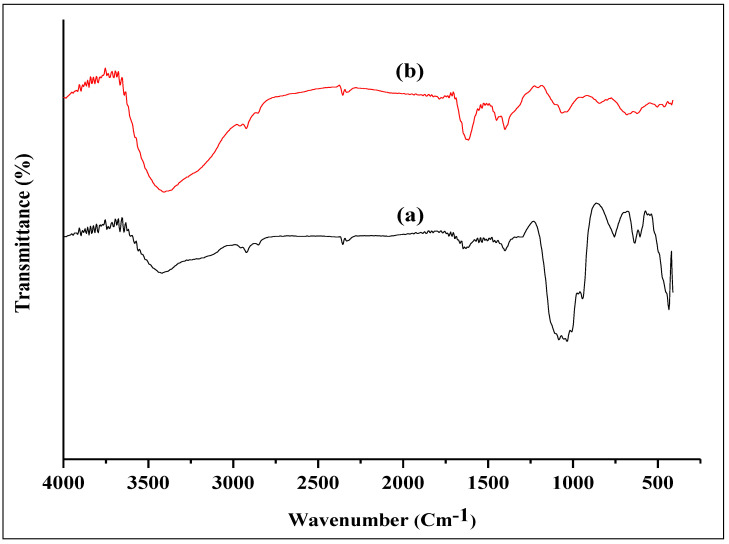
FTIR absorption spectra of (**a**) ZnO NPs and (**b**) ALE of *P. odoratissimum*.

The FTIR technique was used in order to detect possible FGs present in the ALE of *P. odoratissimum* that contribute to the reduction in and stabilization of ZnO NPs. Figure 7a,b represents the FTIR spectra of biosynthesized ZnO nanoparticles and *P. odoratissimum* leaf extract. The peaks of *P. odoratissimum* ALE and biosynthesized ZnO nanoparticles are displayed in Table 3. The broad stretch peak at 3409 cm^−^^1^ and 3417 cm^−^^1^ indicates the presence of an O-H stretch band for the extract and ZnO NPs which are corresponded to the O-H stretching of alcohol, phenolic and flavonoid constituents [61,62]. The low-intensity peaks that arise at 2923 cm^−^^1^ and 2920 cm^−^^1^ were assigned to –CH stretching vibration of the hydroxyl compounds [63,64]. The absorption peaks at 2356 cm^−^^1^ and 2356 cm^−^^1^ were ascribed to O=C=O (stretching vibration) [65]. The peaks observed at 1616 cm^−^^1^ and 1621 cm^−^^1^ indicate the stretching C=C vibration of the aromatic ring system [66,67]. The absorption peaks at 1400 cm^−^^1^ and 1403 cm^−^^1^ correspond to the C-N stretching vibration of amino acids [63]. The strong intensity peaks at 1068 cm^−^^1^ and 1072 cm^−^^1^ are due to the C-O stretching bond of the aromatic rings [67] and may also be related to phenols and flavonoids found in the *P. odoratissimum* ALE in Table 1. The bands at 852 cm^−^^1^ and 855 cm^−^^1^ are attributed to –CH stretching vibration of aromatics [64]. The absorption band observed at 435 cm^−^^1^ confirmed the successful formation of Metal-Oxygen (ZnO). The ZnO absorption peak obtained by FTIR analysis of biosynthesized ZnO NPs has been detected at wavelengths 436 cm^−^^1^ [51], 442 cm^−^^1^ [68], 450 cm^−^^1^ [69] and 485 cm^−^^1^ [70], in the range 400 to 500 cm^−^^1^ [71], which are consistent with our findings. The similarity of bands in both *P. odoratissimum* ALE and *P. odoratissimum*-synthesized ZnO NPs (Table 3) could be attributable to capped biomolecules on the surface of green synthesized ZnO nanoparticles.

#### 3.3.5. X-ray Diffraction (XRD) Analysis of ZnO NPs

**Figure 8:**
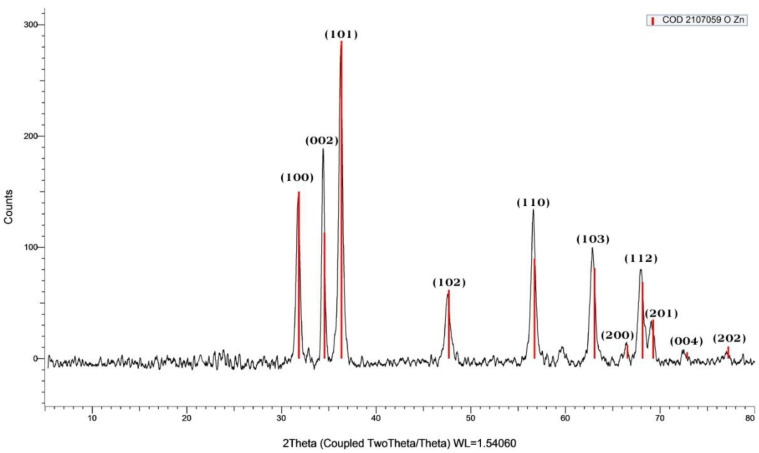
XRD pattern of biosynthesized ZnO NPs via *P. odoratissimum* L. ALE.

The XRD pattern of biosynthesized ZnO NPs using ALE of *P. odoratissimum* is illustrated in Figure 8. The XRD diffraction peaks existed at 2θ angles of 31.85°, 34.55°, 36.35°, 47.69°, 56.75°, 63.09°, 66.56°, 68.17°, 69.29°, 72.87° and 77.21° corresponding to lattice planes (100), (002), (101), (102), (110), (103), (200), (112), (201), (004) and (202), respectively [72]. These peaks are in accordance with those of (JCPDS card No: 36-1451), which is indicating the confirmation of the hexagonal wurtzite structure of ZnO NPs formation [73]. The average crystalline size (ACS) of biosynthesized ZnO NPs was calculated using Deby-Scherrer’s formula [74] and the ACS of the ZnO NPs was estimated to be 14 nm, which is derived from the full width at half maximum (FWHM) of the most intense peak corresponding to (101) plane located at 36.35°. Furthermore, the XRD pattern revealed no additional peaks other than the characteristic ZnO peaks, confirming the purity of the produced ZnO NPs. Additionally, the narrow and strong diffraction peak clearly indicates that the ZnO NPs have an optimal crystalline structure [75,76].

#### 3.3.6. FE-SEM of ZnO NPs

**Figure 9:**
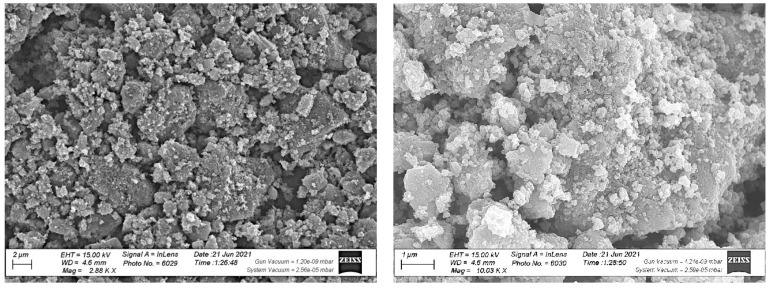

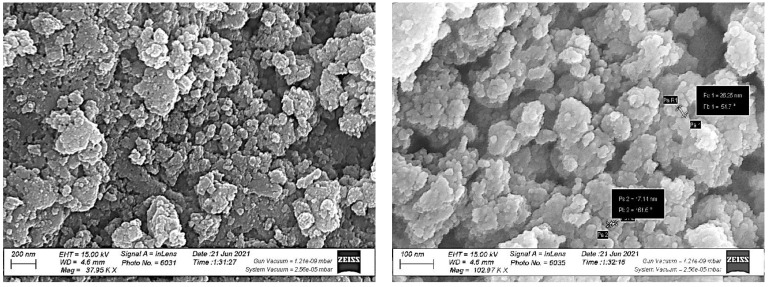
FE-SEM image of biosynthesized ZnO NPs.

**Figure 10:**
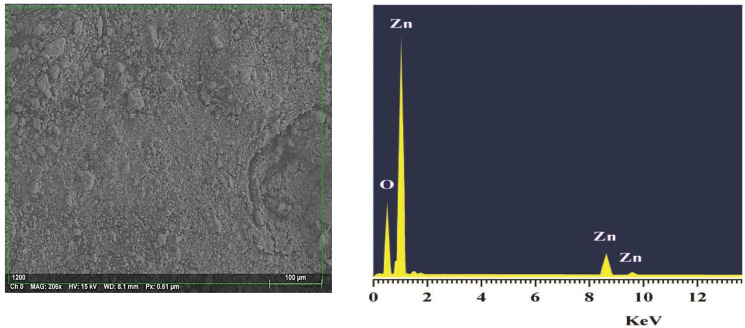
EDX Spectrum of biosynthesized ZnO NPs.

The size and the morphology of the biosynthesized ZnO nanoparticles were imaged via FE-SEM (Figure 9), and the chemical composition of the biosynthesized ZnO nanoparticles was determined using EDX (Figure 10). The FE-SEM image demonstrated that the ZnO NPs were spherical and hexagonal in the morphology shape with good distribution. AN FE-SEM examination showed that the average size of ZnO NPs was 21.6 nm.

#### 3.3.7. Energy Dispersive X-ray Analysis (EDX) Spectrum of ZnO NPs

The elemental mapping of the EDX (Figure 10) verified that the examined sample displayed the elemental peaks of zinc and oxygen which are summarized in Table 4. The EDX analysis proved that the examined sample contained the biosynthesized ZnO NPs.

#### 3.3.8. HR-TEM of ZnO NPs

**Figure 11:**
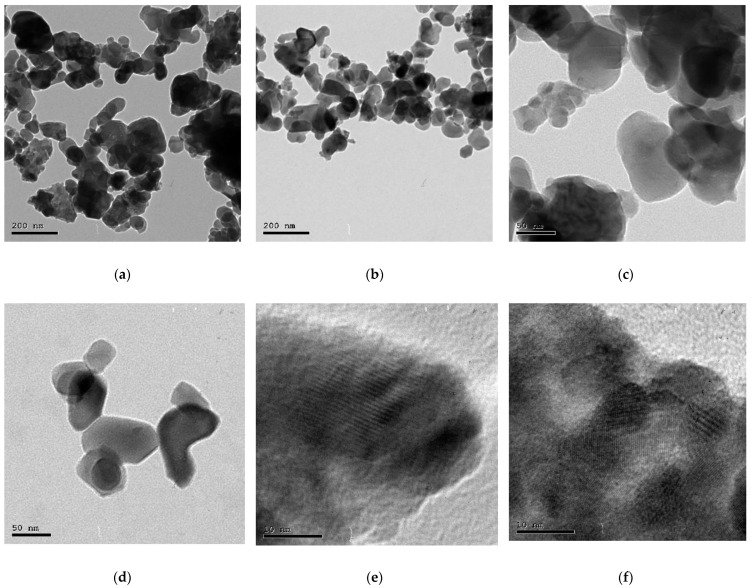

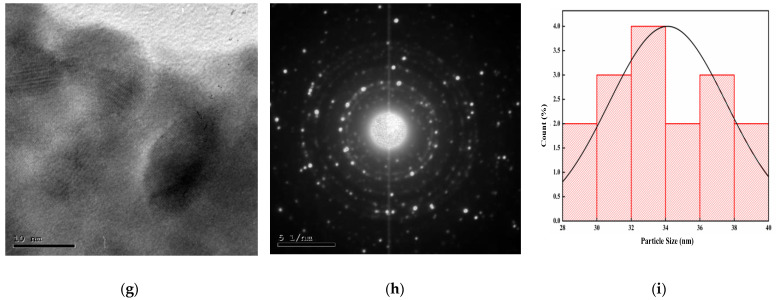
(**a**–**g**) HR-TEM images of biosynthesized ZnO NPs, (**h**) SAED pattern and (**i**) histogram of particle size distribution.

The high-resolution TEM analysis (Figure 11a–g) was carried out to confirm the formation of the biosynthesized ZnO NPs. Based on the results obtained, it can be concluded that the pure green ZnO NPs display hexagonal shapes with an average size of 34.12 nm (Figure 11i) and also clearly reveal lattice fringes without any distortion, indicating that ZnO NPs have high crystallinity. The selected area electron diffraction (SAED) (Figure 11h) pattern revealed a series of rings with bright spots, indicating that ZnO nanoparticles are crystalline in nature [74,76]. Additionally, the hexagonal wurtzite crystalline structure of ZnO NPs is also proven by the diffraction rings on the SAED image and the peaks in the XRD pattern.

### 3.4. Antioxidant Activity

**Figure 12:**
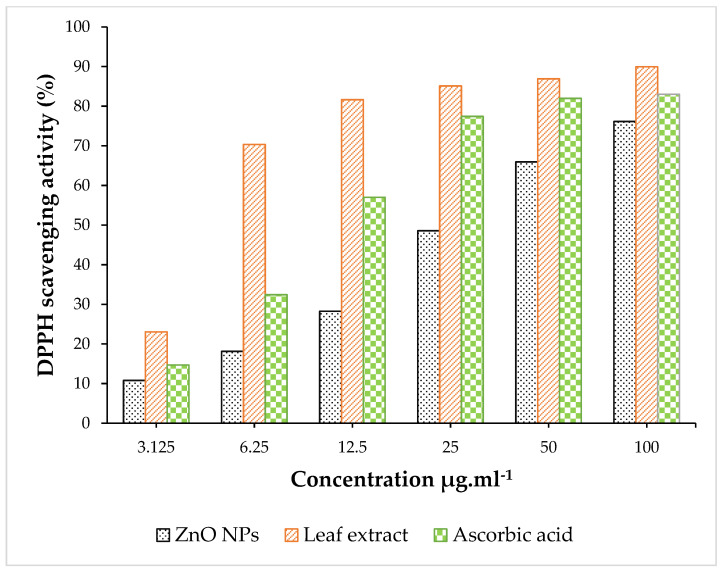
DPPH FRSA of ZnO NPs, ALE and L-ascorbic acid at different concentrations.

The antioxidant activity of ZnO NPs, the ALE of *P. odoratissimum* and L-ascorbic acid are shown in Figure 12. The results obtained show the DPPH scavenging activity of ZnO NPs, ALE and L-ascorbic acid at six different concentrations (3.125 to 100 μg mL^−^^1^) ranging from 10.78 to 76.14%, 23.05 to 89.92% and 14.70 to 83.02% respectively. The DPPH assay showed the scavenging effect of ZnO nanoparticles having an IC_50_ value of 28.11 ± 0.01 μg mL^−^^1^ when compared with the IC_50_ value of L-ascorbic acid (11.50 ± 0.03 μg mL^−^^1^) and aqueous extract (04.56 ± 0.02 μg mL^−^^1^). Additionally, the aqueous extract revealed a superior antioxidant potential to traditional reference L-ascorbic acid, which could be due to various bioactive constituents and the higher content of phenolics and flavonoids present in the *P. odoratissimum* ALE. Moreover, the IC_50_ value of *P. odoratissimum* ALE exhibited higher antioxidants than the aqueous extract of *P. graveolens*, which had an IC_50_ value of 16.59 μg mL^−^^1^ [77].

Generally, phenolic and flavonoid compounds are almost present in all plants in varying proportions and have been reported to act as bio-reductants of metallic ions in an aqueous medium and display a wide range of biological activities such as antioxidant and antimicrobial activity [78]. Many studies have specified that various OH groups’ presence in phenolic and flavonoids are responsible for the formation and stabilization of metal and metal oxide nanoparticles [79,80,81].

As presented in Table 5, the total phenolic content (TPC) of *P. odoratissimum* ALE was found to be 21.93 ± 0.01 mg GAE/g of dried leaf extract, while the total flavonoid content (TFC) was recorded to be 17.11 ± 0.001 mg RE/g of dried leaf extract. From the above results, the ALE of *P. odoratissimum* possesses phytoconstituents that can be used in the formation, capping, stabilization and reduction of zinc acetate salt into ZnO NPs via the green route.

### 3.5. Antibacterial Activity

**Figure 13:**
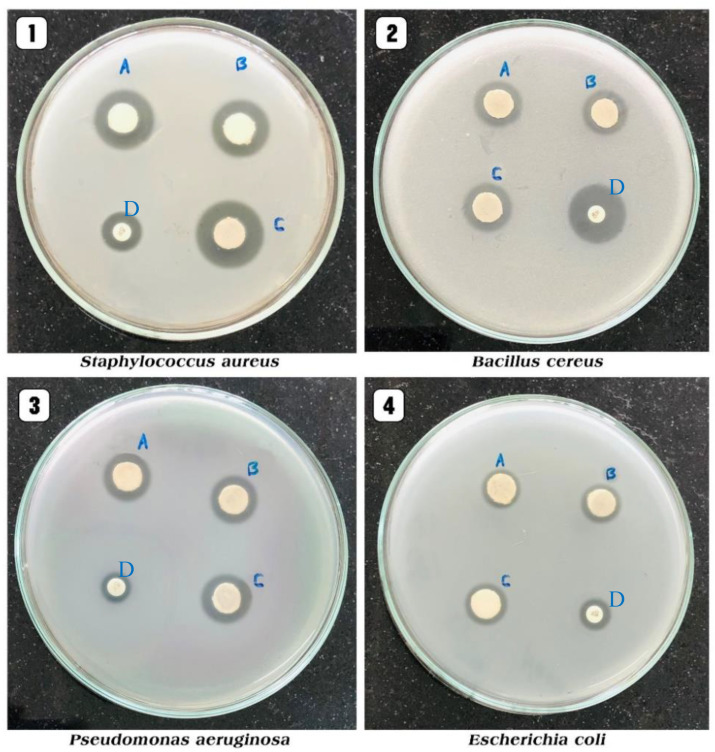
Antibacterial effects (zone of inhibition (mm)) at different concentrations of ZnO NPs (A: 10 μg mL^−^^1^; B: 20 μg mL^−^^1^; C: 30 μg mL^−^^1^ and D: standard) towards various pathogens.

The antibacterial effect of the biosynthesized ZnO NPs was evaluated by disc diffusion assay against *S. aureus* (ATCC 8095), *B. cereus* (ATCC 13753) as GPB, and *E. coli* (ATCC 25922) and *P. aeruginosa* (ATCC10662) as GNB. The results are represented in Table 6 and Figure 13. Generally, the results revealed that the biosynthesized ZnO NPs using *P. odoratissimum* ALE possessed a significant antibacterial effect against all tested bacterial strains. The significant antibacterial zone of inhibition was recorded in *S. aureus* (28 ± 0.35 mm) followed by *B. cereus* (24 ± 0.14 mm), *P. aeruginosa* (21 ± 0.28 mm) and *E. coli* (16 ± 0.21 mm). ALE does not observe any zone of inhibition in the tested bacterial strains. Furthermore, compared to gentamycin as a positive control and ALE of *P. odoratissimum*, biosynthesized ZnO NPs displayed higher antibacterial activity. The antibacterial activities of ZnO NPs differ depending on the cell wall nature of GPB or GNB [82,83]. In the present study, the biosynthesized ZnO NPs showed higher antibacterial activity against GPB (*S. aureus* and *B. cereus*) compared to GNB (*P. aeruginosa* and *E. coli*). A similar trend was obtained by Vijayakumar et al. [10] who stated that ZnO NPs synthesized from *Laurus nobilis* leaf extract displayed greater antibacterial activity against GPB (*S. aureus*) than GNB (*P. aeruginosa*). This is maybe owing to the structure and the components of GPB (i.e., peptidoglycan layer) and may improve the ZnO NPs’ attachment to the cell wall, while the components of GNP avoid this attachment [84].

Additionally, the results indicated that the inhibitory effect of biosynthesized ZnO NPs using *P. odoratissimum* leaf extract increased when the concentration of ZnO NPs was increased. This was in agreement with Gunalan et al. [85], who reported that increasing the concentration of ZnO NPs in discs and wells consistently increased the growth inhibition due to optimal NPs diffusion in the agar medium.

**Figure 14:**
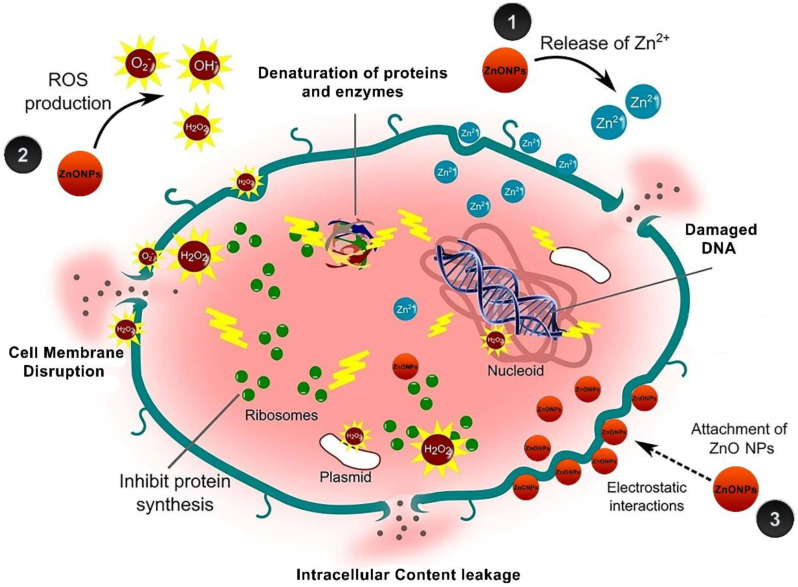
Various proposed mechanisms of ZnO NPs toxicity against bacteria [17].

For the effect of ZnO NPs, there are some proposed bactericidal mechanisms (Figure 14) that have been suggested by scientists. Some suggested that the released Zn from ZnO NPs possess toxic properties that are leading to inhibiting a lot of bacterial cell activities such as bacterial metabolism, and enzyme activity resulting in cell bacterial death [86,87]. The other suggested mechanism is the formation of reactive oxygen species (ROS) that activates oxidative stress which subsequently leads to cell death [88,89]. Another proposed mechanism is the lethal activity of the ZnO NPs due to the attachment of the NPs to the bacterial cell membranes, and the accumulation inside the cytoplasm resulting in damaging the cell membrane integrity and loss of cell contents because of the leakage ending up with cell death [90].

### 3.6. Anti-inflammatory Activity

During times of inflammation, lysosomes lyse and release their component enzymes, resulting in a variety of disorders. Nonsteroidal anti-inflammatory drugs (NSAIDs) work by either blocking lysosomal enzyme release or stabilizing lysosomal membranes [91]. When RBCs are exposed to harmful substances such as hypotonic medium, heat, methyl salicylate (MeS) or phenylhydrazine (PhNHNH_2_), the membranes lyse, resulting in hemolysis and hemoglobin oxidation [92]. Because the membranes of HRBCs are similar to those of lysosomes [91], the inhibition of hypotonicity-induced RBCs membrane lysis was used as a measure of the mechanism of the anti-inflammatory effect of ZnO NPs and *P. odoratissimum* ALE.

From the results obtained in Table 7, the ZnO NPs and *P. odoratissimum* ALE have an anti-inflammatory effect that is concentration-dependent, with the percentage of protection increasing as the concentration of the samples increases. At the concentration of 1000 μg mL^−^^1^, the ZnO NPs significantly (*p* ≤ 0.05) produced 95.60% inhibition of RBC hemolysis, and it was comparable to the results achieved with standard indomethacin (Table 7). The hemolytic effect of the hypotonic solution is due to an excessive accumulation of fluid within the cell, which causes the cell membrane to rupture. Damage to the red cell membrane (RCM) increases the cell’s vulnerability to subsequent damage caused by free radical-induced lipid peroxidation [93]. During a time of increased permeability produced by inflammatory mediators, membrane stability prevents leaking the flow of serum protein and fluids into the tissues [94]. The ZnO NPs and ALE of *P. odoratissimum* maybe stabilized the RBC membrane by preventing the release of active mediators of inflammation and lytic enzymes. Furthermore, many studies have revealed that plant flavonoids have anti-inflammatory and antioxidant activity [95,96,97]. Their anti-inflammatory properties are thought to be owing to an inhibitory action on enzymes involved in the synthesis of the chemical mediators of inflammation and arachidonic acid metabolism [98,99].

## 4. Conclusions

This study presents the biosynthesized ZnO NPs for the first time using an ALE of *P. odoratissimum* via a simple green route. The biosynthesized ZnO NPs showed a characteristic Uv-Vis absorption peak at 370 nm. The XRD pattern also indicated the hexagonal pure Wurtzite structure. FE-SEM coupled with EDX, HR-TEM, FTIR and DLS, confirmed the formation of NPs with an average size of 34.12 nm as obtained from HR-TEM analysis. The DPPH assay revealed that ZnO NPs possess antioxidant activity with an IC_50_ value of 28.11 μg mL^−^^1^. Furthermore, ZnO NPs showed excellent antibacterial effects against both GNB and GPB. In addition, ZnO NPs were found to be more effective as anti-inflammatory via stabilizing the RBCs’ membrane in in vitro models. Our findings suggest the possibility of using the aqueous leaf extract of *P. odoratissimum* for synthesizing stable ZnO NPs. The biosynthesized ZnO NPs possess a significant antioxidant, antibacterial against foodborne pathogenic bacteria and anti-inflammatory activities that can be used as a safe and stable alternative to synthetic substances in the fields of pharmaceutical and biomedical research.

## Figures and Tables

**Table 1:** Qualitative phytochemical screening of *P. odoratissimum* ALE.

Phytoconstituents	Name of Detection Test	Inference
Saponins	Frothing	+
Steroids	Liebermann	−
Triterpenoids	Salkowski	−
Phenolics and tannins	FeCl_3_	+
Flavonoids	Lead (II) acetate	+
Alkaloids	Wagner’s	−
Carbohydrates	Molisch’s	+
Proteins	Biuret	+

(+): present; (−): absent.

**Table 2:** Polyphenolic compounds of *P. odoratissimum* ALE.

Compound	Conc. (µg/g)	Compound	Conc. (µg/g)
Gallic acid	3744.95	Vanillin	0.00
Chlorogenic acid	2523.29	Ferulic acid	2507.38
Catechin	586.08	Naringenin	1870.30
Methyl gallate	63.94	Daidzein	23.74
Caffeic acid	754.77	Quercetin	208.25
Syringic acid	3513.87	Cinnamic acid	11.21
Pyro catechol	0.00	Apigenin	13.56
Rutin	1268.87	Kaempferol	21.50
Ellagic acid	1573.64	Hesperetin	0.00
Coumaric acid	1008.72		

**Table 3:** FTIR spectra of biosynthesized ZnO NPs and *P. odoratissimum* ALE.

Functional Groups	Absorption Bands in ZnO NPs (cm^−1^)	Absorption Bands in *P. odoratissimum* ALE (cm^−1^)
-OH stretch	3417	3409
-C-H stretch	2920	2923
O=C=O stretch	2356	2356
C=C stretch	1621	1616
C-N stretch	1403	1400
C-O stretch	1072	1068
-C-H stretch (aromatics)	855	852
Zn-O	435	-

**Table 4:** Elemental constituents of ZnO NPs.

Element	Weight (%)	Atom (%)
Zn	80.71	50.58
O	19.29	49.42
Total	100	100

**Table 5:** IC_50_, total phenolic (TP), and total flavonoid (TF) contents of *P. odoratissimum* ALE.

Treatment	DPPH IC_50_ (µg/mL)	TPC (mg GAE/g Dry Leaf Extract)	TFC (mg RE/g Dry Leaf Extract)
ALE	04.56 ± 0.02 ^a^	21.93 ± 0.01	17.11 ± 0.001
ZnO NPs	28.11 ± 0.01 ^c^	n.d.	n.d.
L-ascorbic acid	11.50 ± 0.03 ^b^	n.d.	n.d.

n.d. not determined; values expressed as mean of triplicates ± SD (*p* < 0.05). The means of each column with the letters (a–c) differ significantly (*p* < 0.05).

**Table 6:** Evaluation of the antibacterial activity toward pathogenic bacteria.

Pathogenic Bacteria	Diameter of Inhibition Zones (mm)			Positive Control Gentamycin (10 μg mL^−1^)	Aqueous Leaf Extract (20 μg mL^−1^)
	ZnO NPs				
	10 μg mL^−1^	20 μg mL^−1^	30 μg mL^−1^		
*S. aureus*	23 ± 0.70 ^c^	25 ± 1.41 ^b^	28 ± 0.35 ^a^	13 ± 0.28 ^j^	-
*B. cereus*	17 ± 0.35 ^g^	18 ± 0.56 ^f^	24 ± 0.14 ^f^	22 ± 0.70 ^d^	-
*E. coli*	13 ± 0.72 ^j^	15 ± 0.07 ^i^	16 ± 0.21 ^h^	12 ± 0.42 ^k^	-
*P. aeruginosa*	18 ± 1.06 ^f^	20 ± 0.70 ^e^	21 ± 0.28 ^d^	13 ± 0.14 ^j^	-
Mean of ZnO NPs	17.75 ± 3.7 ^C^	19.5 ± 3.5 ^B^	22.25 ± 3.5 ^A^	15 ± 4.00 ^D^	-

Values are means (*n* = 3). According to LSD (as a post hoc test (PHT) at *p* ≤ 0.05), the means of ZnO NPs concentrations sharing different capital letters are significantly different. Interactions between each concentration and bacterial strains are indicated with different superscripted small letters and significantly differ according to LSD as a PHT at *p* ≤ 0.05.

**Table 7:** Effect of the biosynthesized ZnO NPs and ALE of *P. odoratissimum* on hypotonicity-induced hemolysis of HRBCs.

Sample	Conc. (ug/mL)	Mean Absorbance ± SD		Hemolysis Inhibition %
		Hypotonic Solution	Isotonic Solution	
Control		1.326 ± 0.1	0.001 ± 0.01	
ZnO NPs	1000	0.158 ± 0.004 ^b^	0.095 ± 0.00	95.6
	800	0.189 ± 0.003 ^c^	0.071 ± 0.00	91.8
	600	0.264 ± 0.006 ^d^	0.061 ± 0.00	85.9
	400	0.381 ± 0.005 ^f^	0.054 ± 0.00	77.3
	200	0.475 ± 0.002 ^h^	0.035 ± 0.00	69.5
	100	0.583 ± 0.012 ^j^	0.020 ± 0.00	61.0
ALE	1000	0.198 ± 0.007 ^c^	0.081 ± 0.00	91.9
	800	0.329 ± 0.006 ^e^	0.065 ± 0.00	81.7
	600	0.426 ± 0.005 ^g^	0.035 ± 0.00	72.9
	400	0.474 ± 0.007 ^h^	0.031 ± 0.00	69.3
	200	0.544 ± 0.005 ^i^	0.027 ± 0.00	64.1
	100	0.660 ± 0.003 ^k^	0.022 ± 0.00	55.7
Indomethacin	1000	0.059 ± 0.002 ^a^	0.035 ± 0.01	98.1

Values are expressed as the mean of triplicates ± SD. Different superscripted small letters significantly differ based on LSD as a post hoc test at *p* ≤ 0.05.

## Data Availability

The data presented are included within the article.
